# Non-medical use of opioids among HIV-infected opioid dependent individuals on opioid maintenance treatment: the need for a more comprehensive approach

**DOI:** 10.1186/1477-7517-8-31

**Published:** 2011-11-28

**Authors:** Perrine Roux, Patrizia M Carrieri, Julien Cohen, Isabelle Ravaux, Bruno Spire, Michael Gossop, Sandra D Comer

**Affiliations:** 1 INSERM, U912 (SE4S), 23 rue Stanislas Torrents, 13006 Marseille, France; 2 Université Aix Marseille, IRD, UMR-S912, Marseille, France; 3 ORS PACA, Observatoire Régional de la Santé Provence Alpes Côte d'Azur, Marseille, France; 4 Hôpital La Conception, Service des Maladies Infectieuses, 147 boulevard Baille, 13005 Marseille, France; 5 King's College London, 4 Windsor Walk, London, SE5 8BB, UK; 6 Division on Substance Abuse, New York State Psychiatric Institute and Columbia University, NY, USA

**Keywords:** opioid maintenance treatment, buprenorphine, methadone, non-medical use, HIV, withdrawal, antiretrovirals

## Abstract

**Background:**

Opioid maintenance treatment (OMT) has a positive impact on substance use and health outcomes among HIV-infected opioid dependent patients. The present study investigates non-medical use of opioids by HIV-infected opioid-dependent individuals treated with buprenorphine or methadone.

**Methods:**

The MANIF 2000 study is a longitudinal study that enrolled a cohort of 476 HIV-infected opioid-dependent individuals. Data were collected in outpatient hospital services delivering HIV care in France. The sample comprised all patients receiving OMT (either methadone or buprenorphine) who attended at least one follow-up visit with data on adherence to OMT (N = 235 patients, 1056 visits). Non-medical use of opioids during OMT was defined as having reported use of opioids in a non-medical context, and/or the misuse of the prescribed oral OMT by an inappropriate route of administration (injection or sniffing). After adjusting for the non-random assignment of OMT type, a model based on GEE was then used to identify predictors of non-medical use of opioids.

**Results:**

Among the 235 patients, 144 (61.3%) and 91 (38.9%) patients were receiving buprenorphine and methadone, respectively, at baseline. Non-medical use of opioids was found in 41.6% of visits for 83% of individual patients. In the multivariate analysis, predictors of non-medical use of opioids were: cocaine, daily cannabis, and benzodiazepine use, experience of opioid withdrawal symptoms, and less time since OMT initiation.

**Conclusions:**

Non-medical use of opioids was found to be comparable in OMT patients receiving methadone or buprenorphine. The presence of opioid withdrawal symptoms was a determinant of non-medical use of opioids and may serve as a clinical indicator of inadequate dosage, medication, or type of follow-up. Sustainability and continuity of care with adequate monitoring of withdrawal symptoms and polydrug use may contribute to reduced harms from ongoing non-medical use of opioids.

## Background

Among HIV-infected opioid dependent individuals, the clinical management of drug dependence is a matter of great concern. This issue is especially relevant in those countries where the HIV epidemic is driven by injecting drug users (IDUs) [1,2]. Even in industrialized countries, HIV-infected opioid-dependent persons seeking care for their drug dependence may face many barriers to effective treatment, and their management may be complicated by the difficulties associated with the provision of multiple treatments [3]. Opioid maintenance treatment (OMT) has been found to reduce high risk behaviors related to HIV transmission such as injecting drugs, sharing needles/syringes, and having unprotected sex [4]. In France, two forms of OMT, with buprenorphine and methadone, are available and provision of these treatments has been found to have a substantial beneficial impact upon the growth of the HIV epidemic [5].

The initiation of OMT with methadone or buprenorphine in HIV-infected opioid dependent patients has been found to have a positive impact on health outcomes [6], and plays an important role in sustaining adherence to antiretroviral treatment (ART) for HIV infection [7]. The topic of adherence to ART has been widely studied since the beginning of the HIV epidemic [8]. In injecting drug users (IDUs) [9], adherence to ART is important because sub-optimal adherence to ART may lead to the risk of HIV resistance and accelerated progression of disease [10]. In this paper, we focused on non-medical use of opioids defined as either use of illicit opioids such as heroin or other non-prescribed opioids, or use of OMT (buprenorphine or methadone) by a non medically prescribed route of administration. Non-medical use of opioids, especially by injection, is particularly relevant in HIV-positive patients because it is a major correlate not only of response to OMT but also to antiretroviral therapy response as expressed by non-adherence [7] and virological failure [11].

The MANIF 2000 cohort study took place in several settings in France and was designed to focus on socio-behavioral aspects of HIV-positive IDUs, with particular emphasis on their access and adherence to antiretroviral treatment as well as OMT-related outcomes. The inclusion of HIV-infected opioid-dependent individuals, while buprenorphine and methadone were launched to treat opioid drug dependence, provided us with the opportunity to identify the correlates of non-medical use of opioids during opioid maintenance treatment.

## Methods

### Cohort and sample

The French MANIF 2000 cohort in 1995/1996 enrolled 467 patients who were HIV-positive. Inclusion criteria for enrollment in the cohort were: receiving OMT treatment, patients with a CD4+ cell count > 300 during the last visit prior to enrolment and in clinical stage A or B. This cohort was designed to focus on social and behavioral aspects of HIV-positive IDUs and particularly on their access [12] and adherence to antiretroviral treatment [13] as well as to OMT [14]. In this study, we only selected visits during time periods when patients were enrolled in OMT, either methadone or buprenorphine, and had available data on opioid use, including OMT. The sample comprised 235 individuals, accounting for a total of 1056 visits. All individuals who agreed to be interviewed signed an informed consent form, approved with the study protocol by the Committee for the Protection of Persons (CPP) involved in biomedical research.

### Data collection

Data were collected at 6-month intervals by means of a face-to-face interview, a self-administered questionnaire, and medical records. The face-to-face interview was based on a standardized protocol, administered by trained nurses, which gathered psychosocial information and patients' personal experience with HIV infection and care. The self-administered questionnaire included socio-demographic data, prison history, substance use and related behaviours, OMT exposure (methadone or buprenorphine), and consumption of psychotropic drugs and alcohol during the previous 6 months. Self-reported use of heroin and morphine were checked for validity at enrollment by morphine detection in urine samples. Injection drug use at any given visit thereafter was defined as the injection of any drug in the 6 months before that visit.

Clinical and laboratory data on viral load, CD4 T-cell counts and data on HIV clinical stage, with stage C indicating progression to AIDS disease were collected every 6 months from the physician or from medical records [15]. An undetectable viral load was defined as an HIV-1 RNA level below the lower limit of detection of the assay and was considered a virological success. Information was also collected about the first positive HIV test and patients' ART history.

Depression was measured using the French version of the Center for Epidemiological Studies Depression Scale (CES-D) [16]. The 75th percentile (age-gender specific) of the distribution of values for the corresponding indicator in the general French population was used to classify patients as depressed or not depressed at each interview. Then, the CES-D score was dichotomized by using 17 and 23 as cut-off points for men and women, respectively, as indicative of depression, on a score scale ranging from 0 to 60, as already validated in a previous study [17].

Involvement with "non-medical use of opioids" was collected through both patients' and physicians' answers to the questionnaires. There was little discrepancy between physicians' and patients' reports about OMT: in cases of disagreement, patients' self reports were considered as more reliable and were used in the analyses. At any given visit, time since OMT initiation was computed as the uninterrupted time-interval between the last initiation or re-initiation of OMT until that visit. Also, the prescribed dose of OMT was considered low if the methadone dose was less than 60 mg per day or the buprenorphine dose was less than 8 mg per day.

Information about the use of opioids and OMT was collected by means of the self-administered questionnaire and the structured face-to-face interview. Non-medical use of opioids was defined as use of illicit or non-prescribed opioids, and/or the misuse of the prescribed OMT by using an inappropriate route of drug administration. Specifically, individuals were considered as having non-medical use of opioids if at any given visit they reported use of any opioid drug other than their prescribed OMT medication, or if they reported having administered their prescribed OMT by sniffing or injection in the previous 6 months.

### Statistical analysis

A 2-step Heckman approach allowed us to account for the non-random assignment to different types of OMT medication. The first step, explained in a previous paper [18], was based on a probit model to identify predictors of starting either buprenorphine or methadone treatment and led to the computation of the inverse Mills ratio (IMR). The IMR was then introduced in the second step model to correct for the potential non-random assignment of buprenorphine and methadone (prescription bias).

A model based on Generalized Estimating Equations (GEE) was then used to identify predictors of non-medical use of opioids while correcting for the bias induced by non-random assignment. Variables with p-values < 0.20 in the univariate analysis of each step were considered eligible for inclusion in the multivariate models. An exchangeable correlation matrix was used for the GEE models. All variables tested, including the IMR, were considered eligible to enter the model. A backward procedure was used to identify the best GEE model and variables were removed one at a time based on a p-value > 0.05. The log-likelihood ratio test was used to identify the best pattern of predictors. Bias-corrected confidence intervals and p-values were based on 400 bootstrap replications.

## Results

### Descriptive results at baseline

Among the 235 patients, 163 (69.4%) were men and 72 (30.6%) were women. The median [IQR] age was 34 [31-37] years. Thirty-four (14.8%) patients had a high school certificate and 150 (63.8%) were the owner or renter of their accommodation. Depressive symptoms were found in 170 (73%) patients. Use of cocaine was reported by 85 (36.5%) and heroin use by 141 (60.5%). One hundred and forty eight (63%) patients reported having injected any drug in the 6 months before the first visit on OMT. Heavy drinking was reported by 57 (24.9%) patients. Buprenorphine was the more commonly prescribed type of medication: this was prescribed to 144 (61.3%) patients at baseline, with 91 (38.9%) receiving methadone. About one fifth of the patients (n = 46, 19.6%) were being treated with ART.

### Descriptive results during the study period

The descriptive analysis of the whole study period is presented in Table 1.

**Table 1:** Factors associated with non-medical use of opioids during opioid maintenance treatment: univariate analyses.

	Number of visits (%) or median [IQR]	Nb. of patients	Coefficient (95% CI)	p-value
Buprenorphine (ref)	640 (60.6)	154		
Methadone	416 (39.4)	111	0.00 (-0.41; 0.37)	0.98
Female gender	286 (27.1)	72	0.16 (-0.09; 0.39)	0.20
Age^a^	36 [32-39]		**-0.69 (-0.96; -0.43)**	**< 10^-3^**
Owner or renter of their house	757 (71.7)	189	**-0.24 (-0.45; 0.00)**	**0.03**
Good housing conditions^b^	868 (82.4)	210	-0.13 (-0.39; 0.08)	0.22
Heavy drinking^c^	213 (20.4)	95	0.15 (-0.07; 0.40)	0.20
Cocaine use^d^	277 (26.3)	115	**0.91 (0.67; 1.20)**	**< 10^-3^**
Cannabis use (daily)^d^	467 (44.4)	156	**0.45 (0.29; 0.62)**	**< 10^-3^**
Benzodiazepine consumption^d^	358 (33.9)	150	**0.62 (0.44; 0.81)**	**< 10^-3^**
HIV clinical stage				
A (ref)	474 (44.9)	138		
B	518 (49.1)	125	**-0.24 (-0.49; -0.01)**	**0.06**
C	63 (6.0)	22	**-0.68 (-1.17; -0.28)**	**0.003**
Depressive symptoms^e^	666 (63.2)	200	**0.34 (0.18; 0.52)**	**< 10^-3^**
Withdrawal symptoms	204 (19.3)	122	**0.81 (0.56; 1.07)**	**< 10^-3^**
Time since first injection (years)^a^	16 [13-20]		**-0.56 (-0.86; -0.32)**	**< 10^-3^**
Time since first positive HIV test (years)^a^	11 [8-13]		**-1.00 (-1.35; -0.76)**	**< 10^-3^**
Time since OMT initiation (months)^f^	16 [7-30]		**-0.29 (-0.39; -0.22)**	**< 10^-3^**
Undetectable viral charge	366 (34.9)	124	**-0.25 (-0.46; -0.03)**	**0.03**
CD4 cell count/mm^3 ^< 200	57 (5.5)	32	-0.26 (-0.65; 0.15)	0.20
Receiving ART	395 (37.4)	119	**-0.52 (-0.70; -0.30)**	**< 10^-3^**
Complete trust in physicians	824 (78.4)	196	**-0.33 (-0.62; 0.10)**	**0.01**
Low prescribed dose of OMT^g^	676 (66.8)	169	-0.22 (-0.43;0.04)	0.06

*MANIF 2000 cohort, N = 235, 1056 visits.

^a ^For ten years increase.

^b ^Good housing conditions were defined as the rank 3 and 4 (quite or very comfortable vs. uncomfortable or low comfort) using a four-point Likert scale.

^c ^Heavy drinking was defined as drinking more than 4 alcohol units on any one occasion.

^d ^During the previous 6 months.

^e ^Patients were defined with depressive symptoms if CES-D > 17 for men and > 23 for women.

^f ^For one year increase.

^g^Prescribed dose of OMT is low if the methadone dose was less than 60 mg per day or the buprenorphine dose was less than 8 mg per day.

During the study period, the median [IQR] time since the initiation of opioid maintenance treatment was 16 [13-20] months. Among the 235 patients, 18 switched from buprenorphine to methadone, 10 switched from methadone to buprenorphine, and one switched from buprenorphine to methadone and then back to buprenorphine. Among all the treatment visits, non-medical use of opioids was found in 439 (41.6%) visits for 196 individual patients. In addition, the mean duration of study follow-up for our sample was 45 months.

### First step: Predictors of OMT prescription, buprenorphine or methadone

In the first step model, factors independently associated with methadone prescription were unemployment, drinking more than 4 units of alcohol per day, cocaine use in the 6 months prior to the visit, and smoking more than 20 cigarettes per day [18].

### Second step: Univariate and multivariate analyses

The results of the univariate analysis are presented in Table 1. No statistically significant difference was found between type of maintenance medication (buprenorphine or methadone) with regard to non-medical use of opioids, after correcting for the bias induced by non-random assignment. Regarding HIV status, a number of variables were found to be associated with non-medical use of opioids in the univariate but not in the multivariate analyses: not receiving ART, being at clinical stage A (compared to B and C), having a higher duration since first testing positive for HIV, not having an undetectable viral charge and not reporting having a complete trust in the physician. Socio-demographic factors that were found to be associated with non-medical use of opioids included younger age, and not being owner or renter of his/her accommodation. Patients who reported depressive symptoms were more likely to have reported non-medical use of opioids. Lower doses of OMT were not significantly associated with non-medical use of opioids.

In the multivariate analysis presented in Table 2 five variables that were found to be significant in the univariate analysis remained significantly associated with the outcome. These included non-opioid drug use (use of cocaine and benzodiazepines, and daily use of cannabis). Patients who reported having experienced opioid withdrawal symptoms in the 6 months prior to the visit were found to report more non-medical use of opioids. Uninterrupted attendance at an OMT program until a given visit was associated with less non-medical use of opioids.

**Table 2:** Factors associated with non-medical use of opioids during opioid maintenance treatment: multivariate analysis.

	Adjusted coefficient (95% CI)	p-value
Cocaine use^d^	0.81 (0.57; 1.10)	< 10^-3^
Cannabis use (daily)^d^	0.28 (0.08; 0.47)	0.01
Benzodiazepines consumption^d^	0.37 (0.16; 0.55)	< 10^-3^
Withdrawal symptoms	0.62 (0.36; 0.89)	< 10^-3^
Time since OMT initiation (months)^f^	-0.25(-0.35;-0.18)	< 10^-3^

^d ^During the previous 6 months.

^f ^For one year increase.

## Discussion

Non-medical use of opioids was found to be comparable in HIV-infected opioid dependent patients receiving methadone or buprenorphine. In addition, our results showed that ongoing use of non-opioids (such as cocaine, cannabis, and benzodiazepines), perception of withdrawal symptoms, and a shorter retention in OMT are associated with non-medical use of opioids. At a time when non-medical use of prescription opioids [19] and use of opioids by injection [20-22] are growing problems and a real concern for public health, identifying correlates of non-medical use of opioids is of interest and should help physicians to better manage opioid dependence and improve the effectiveness of OMT in not only HIV-infected population but also the whole population of opioid-dependent individuals.

First, it is interesting to note that, according to the results of the univariate analysis, HIV care has a positive impact on decreasing non-medical use of opioids. Patients who were receiving antiretroviral treatment (ART) were less likely to report non-medical use of opioids. This result may be related to the wider access to ART in IDUs who achieved stabilization on OMT [23]. Moreover, it is known that access to ART has a positive impact on drug injecting cessation in HIV-infected IDUs [24]. This impact could be explained by the reality of the relationship between physician and patient based upon trust in the ART initiation process [25], as suggested in our univariate analysis. This latter result supports the idea that access to ART in HIV-infected opioid dependent individuals could play a harm reduction role.

However, the results of the multivariate analysis show the most important predictors of non-medical use of opioids which are not related to HIV care in our analysis. And after multiple adjustments (including for non random assignment of buprenorphine and methadone), only shorter time since OMT initiation, experience of withdrawal symptoms, and non-opioid drug use were found to be significant determinants of non-medical use of opioids. Among the independent predictors of non-medical use of opioids, some patients' illicit substance use behaviors such as cocaine, daily cannabis, and benzodiazepine use were associated with the outcome. These findings are consistent with those of previous studies showing that non-opioid drug use in OMT-treated patients may negatively influence treatment outcomes. For instance, cocaine use during OMT has previously been shown to be a predictor of buprenorphine diversion by injection in patients receiving prescribed buprenorphine [14] and to hinder efficacy of OMT [26]. In addition, OMT patients who had used benzodiazepines were more likely to have opioid-positive urine screens during OMT [27]. This is a concern because few guidelines are available to physicians who are required to treat co-dependent drug users. A recent meta-analysis has summarized the most common approaches to treating benzodiazepine dependence [28] and the most effective is a combination of a gradual dose reduction regimen and psychological interventions. Moreover, a recent study showed that opioid dependent individuals receiving heroin-assisted treatment were more likely to decrease their benzodiazepine use compared to those receiving methadone maintenance treatment [29]. Finally, heavy cannabis use also has been found to be correlated with buprenorphine injection [30]. Polydrug use should be considered more thoroughly not only for measuring the independent effects of multiple drug use but also for investigating the effects of drug use patterns on behavioural outcomes [31].

Although the association between time since OMT initiation and non-medical use of opioids is not unexpected, it demonstrates that the longer the duration of OMT (retention in OMT), the better achievement of stabilization in terms of heroin use and OMT diversion [32]. The clinical management of opioid dependence has already been described as a very long process with a high risk of cycling in and out of treatment [33].

Patients who reported withdrawal symptoms during the study period were also found to report higher non-medical use of opioids. This result is clinically very relevant and suggests that physicians should investigate subjective and objective withdrawal symptoms during treatment in order to better understand the reasons that inter-dose opioid withdrawal exist [34]. Their occurrence may be indicative of inadequate OMT dosage [35,36], either too low, which leads to withdrawal symptoms directly, or too high, which leads to aversive side effects that lead to discontinuation of OMT. However, the results do not support the notion that lower doses of OMT are related to more non-medical use of opioids, probably because low doses were a proxy for less severe opioid dependence and not for inadequate dose prescription. Other factors could be related to disliking of the subjective effects of OMT, or polydrug use, which also may lead patients to interrupt treatment [37,38]. This issue is all more relevant since Mateu-Gelabert et al showed that withdrawal symptoms could increase risky behaviors regarding HIV and also Hepatitis C virus (HCV) transmission by undermining IDUs' willingness to inject safely [39].

Recent WHO guidelines recommend that the dose of OMT should be increased progressively according to clinical effect in the individual patient, up to an adequate dosage [40]. In addition, drug interactions represent a potential concern in HIV-infected individuals, especially with regard to methadone and protease inhibitors, and in these circumstances, methadone dose adjustments may be required to avoid withdrawal symptoms [41]. In addition, the experience of withdrawal symptoms among HIV-infected patients who are receiving OMT needs careful surveillance and clinical management since the presence of withdrawal symptoms has been associated with an increased risk of mortality in the same cohort [42].

After adjustment for non-random allocation to buprenorphine/methadone prescription, we found no difference between methadone and buprenorphine with regard to non-medical use of opioids. These findings are consistent with previous studies showing that methadone and buprenorphine are broadly equivalent at higher dosages [43]. The correction for the bias elicited by non-random assignment was necessary due to the two different models of OMT delivery in France which may lead to the selection of different opioid drug dependent populations. Historically, in France, initiation of methadone is possible only in specialized centers for drug dependence while access to buprenorphine is available in primary care settings [5]. This differential access could explain why methadone in France is more frequently initiated in patients presenting a severe addictive profile. In France, methadone programs provide multidisciplinary care, whereas buprenorphine is delivered through primary care settings, which have minimal ancillary services. Previous research has shown that retention in OMT is influenced by several in-treatment variables such as occurrence of depressive symptoms, social functioning [44] and trust in the physician [45]. Therefore, although more severely dependent patients were included in the methadone group, it is not entirely surprising that non-medical use of opioids did not differ from the buprenorphine group in our study because the methadone patients had access to more comprehensive care [46].

Some limitations of this study should be acknowledged. Data on substance use was based on self-reported behaviors, which are often questioned due to possible social desirability bias. However, the validity and reliability of self-reports about active drug use have been established in many studies that used similar methods for collecting information about substance use behaviors [47,48] as well as in a previous study with a sample of HIV-infected patients in which substantial agreement was documented between self-reported heroin use and morphine detection in urine [49]. Our results do not allow a clear inference about the direction of effects. Although the study started some years ago, this cohort remains a very interesting observational sample of patients, especially for countries that have only recently started to scale up antiretrovirals and OMT for opioid dependent HIV-infected individuals. Finally, although a statistical adjustment was made to allow for non-random assignment to medication type, this study was not a controlled clinical trial and further investigations are required to confirm our findings.

## Conclusions

The results of the present study contribute towards a better understanding of the factors associated with non-medical use of opioids, and may also have implications for understanding adherence to anti-retroviral therapy in HIV-infected opioid-dependent individuals. When methadone and buprenorphine are effective in stabilizing opioid dependence, they can also improve health and socio-economic outcomes in HIV positive IDUs [50]. Clinically, these findings demonstrate the important relationship between withdrawal symptoms in OMT treated patients and positive response to medication. More generally, it emphasizes the importance of being attentive to the provided care and to the environment of patients. These findings corroborate the idea that stabilization on OMT is not only a patient problem [51]. Our data suggest that some combined and novel approaches are necessary to decrease the harms associated with abuse or dependence on benzodiazepines, cocaine, and/or cannabinoids, especially in HIV-infected individuals. Clinical intervention studies aimed at optimizing effectiveness of OMT in HIV-infected patients are required to better understand the extent of a more comprehensive approach combining attention to withdrawal symptoms, and treatment for other drug dependence. Programs with better patients retention can improve adherence to ART and HIV outcomes in people living with HIV. Further research on new medications and new interventions for co-dependence on stimulants and/or benzodiazepines is required to develop a more comprehensive and effective model of care for such populations and to assure improved OMT and HIV outcomes.

## List of Abbreviations

ANRS: Agency for Research on Aids and Viral Hepatitis; ART: Antiretroviral Treatment; CD4: Cluster of Differentiation 4; CES-D: Center for Epidemiological Studies Depression Scale; CPP: Committee for the Protection of Persons; GEE: Generalized Estimating Equations; HCV: Hepatitis C Virus; HIV: Human Immunodeficiency Virus; IDUs: Intravenous Drug Users; IQR: Interquartile Range; OMT: Opioid Maintenance Treatment; OR: Odds Ratio; RNA: RiboNucleic Acid.

## Competing interests

Dr. Comer has received research grant funding from NIDA (DA09236, DA10909), as well as unrestricted educational grants from Reckitt Benckiser and Schering Plough to study the abuse liability of buprenorphine. The other authors who have taken part in this study declared that they do not have anything to disclose regarding funding or conflict of interest with respect to this manuscript.

## Authors' contributions

PMC, IR and BS were involved in the study concept and design as well as the acquisition of data. Statistical analyses and interpretation of data were performed by PMC, JC, SDC, MG, PR and BS. PR was principally involved in the drafting of the manuscript under the supervision of PMC and SDC. All authors read and approved the final manuscript.
